# Laser-induced thermal grating spectroscopy based on femtosecond laser multi-photon absorption

**DOI:** 10.1038/s41598-021-89269-2

**Published:** 2021-05-10

**Authors:** Maria Ruchkina, Dina Hot, Pengji Ding, Ali Hosseinnia, Per-Erik Bengtsson, Zhongshan Li, Joakim Bood, Anna-Lena Sahlberg

**Affiliations:** 1 Physics Department, Lund University, P.O. Box 118, 221 00 Lund, Sweden; 2 School of Nuclear Science and Technology, Lanzhou University, 222 Tianshui S Rd, Lanzhou, China

**Keywords:** Applied physics, Atomic and molecular interactions with photons, Electronic structure of atoms and molecules, Optical spectroscopy, Nonlinear optics, Ultrafast photonics

## Abstract

Laser-induced grating spectroscopy (LIGS) is for the first time explored in a configuration based on the crossing of two focused femtosecond (fs) laser pulses (800-nm wavelength) and a focused continuous-wave (cw) laser beam (532-nm wavelength). A thermal grating was formed by multi-photon absorption of the fs-laser pulses by $\hbox {N}_{{2}}$ with a pulse energy around 700 $\upmu$J ($\sim$ 45 TW/$\hbox {cm}^{2}$). The feasibility of this LIGS configuration was investigated for thermometry in heated nitrogen gas flows. The temperature was varied from room temperature up to 750 K, producing strong single-shot LIGS signals. A model based on the solution of the linearized hydrodynamic equations was used to extract temperature information from single-shot experimental data, and the results show excellent agreement with the thermocouple measurements. Furthermore, the fluorescence produced by the fs-laser pulses was investigated. This study indicates an 8-photon absorption pathway for $\hbox {N}_{{2}}$ in order to reach the $\hbox {B}^{3}\Pi _{g}$ state from the ground state, and 8 + 5 photon excitation to reach the $\hbox {B}^{2}\Sigma _{u}^{+}$ state of the $\hbox {N}_{2}^{+}$ ion. At pulse energies higher than 1 mJ, the LIGS signal was disturbed due to the generation of plasma. Additionally, measurements in argon gas and air were performed, where the LIGS signal for argon shows lower intensity compared to air and $\hbox {N}_{{2}}$.

## Introduction

Reliable non-intrusive methods for gas-phase diagnostics are of great value for energy and combustion science^1^. Even for the simplest fuels, the combustion process often involves more than 100 elementary reactions and a large number of species. In order to probe such processes, proper analytical techniques with a sufficient degree of spatial and temporal resolution should be employed. These techniques should at the same time not alter the chemistry and gas flow, and since many species are unstable, measurements should be performed in-situ. Several different laser-based diagnostic techniques have been developed, e.g. for species concentration, temperature, velocity and particle measurements, in order to meet these high demands^1–3^.

Laser-induced grating spectroscopy (LIGS) is a technique used for gas phase diagnostics, where various thermodynamic properties of the medium can be inferred such as temperature^4–6^, thermal diffusivity^7^ and, under favourable conditions, the species concentrations^8^. In a typical LIGS experiment, two coherent pump beams from the same pulsed laser source are crossed and create an interference pattern in the intersection region. A laser-induced grating (LIG) can be formed by two different mechanisms: (1) *thermalization* of the absorbed laser radiation by absorbing species in the medium via collisions, which results in modulation of the gas density and thus modulation of refractive index, and (2) *electrostriction* that results in a modulation of the gas density caused by compression of the molecules due to the strong electric fields of the pump laser light^9^. The formation and dynamics of the LIG can be probed by aligning a long-pulse or continuous-wave (cw) laser beam to intersect the LIG at Bragg angle. A small fraction of the probe beam will be diffracted by the LIG, which forms the signal. Unlike many other diagnostic techniques, LIGS has shown great potential for applications in high pressure environments^5^, where the signal intensity increases with increasing pressure. Both thermal and electrostrictive LIGS have been applied for temperature measurements in heated gas^4,10–12^, in flames^4,13–16^ and in engines^17–19^.

To date most LIGS methods for thermometry are based on nanosecond lasers which are tuned to a specific wavelength for direct resonant absorption. Fourkas et al.^20,21^ used picosecond laser pulses to perform LIGS measurements in a $\hbox {CH}_{{4}}$/air flame seeded with sodium. In that work, the wavelength of the picosecond pulsed laser was tuned to the sodium D-lines. During the last decades, an increasing number of lasers operating with shorter pulses in the femtosecond regime have become commercially available. The use of ultrashort intense laser pulses enables efficient excitation to high-lying energy states through multi-photon absorption, and thus providing access to atoms/molecules with resonances in the vacuum ultraviolet (VUV) regime (e.g. $\hbox {N}_{{2}}$). The ultrashort pulse duration offers efficient pumping even with relatively low pulse energies, thereby decreasing the probability of photodissociation processes^22^.

The primary goal of the current study is a demonstration of the feasibility of femtosecond (fs)-LIGS as a measurement technique for both fundamental studies and remote non-intrusive diagnostics of various gaseous environments. For this purpose, the simple diatomic molecule $\hbox {N}_{{2}}$ was selected as absorbant for LIGS thermometry due to its high abundance in air-fed combustion processes. The excitation of $\hbox {N}_{{2}}$ with high intensity ultrashort laser pulses might result in irradiances in the probe volume high enough for ionization, laser trapping^23^, and “lasing” actions^24^. However, in the present study, the pulse energy was kept below the ionization threshold in order to avoid additional uncertainties in the measurement results.

In this work we started with studying the possible absorption pathways in molecular nitrogen. A spectroscopic investigation of the fluorescence originating from the transitions $\hbox {C}^{3}\Pi _{u}$ $\rightarrow$ $\hbox {B}^{3}\Pi _{g}$ and $\hbox {B}^{2}\Sigma _{u}^{+}$ $\rightarrow$ $\hbox {X}^{2}\Sigma _{g}^{+}$ of $\hbox {N}_{{2}}$ and $\hbox {N}_{2}^{+}$, respectively, was undertaken. This study revealed the vibronic states populated by multi-photon absorption. We have performed theoretical simulations in PGOPHER^25^ in order to confirm the experimentally observed fluorescence from $\hbox {N}_{{2}}$ and $\hbox {N}_{2}^{+}$. However, the main focus of the paper is the study of LIGS signals, which have been recorded at different temperatures, ranging from room temperature up to 753 K. A LIGS signal model has been fitted to the measured signals in order to extract the gas temperature. This investigation indicates a promising potential for fs-LIGS thermometry.

## Methods

Two crossed pump beams from the same pulsed laser source create a grating, i.e. a spatial modulation of the refractive index, which is probed by a third laser beam. The crossing of the two pump beams results in the production of interference fringes with a spatial period

1 $$\begin{aligned} \Lambda = \frac{\lambda _{pump}}{2\sin \left( \frac{\theta }{2}\right) }, \end{aligned}$$

where $\lambda _{pump}$ is the wavelength of the pump laser, and $\theta$ is the crossing angle. When the pump laser wavelength is in resonance with an absorbing atom/molecule in the probe volume, it will be pumped into an excited state.

The subsequent collisional relaxation of the excited species results in heat release, which occurs along the high intensity fringes. This results in the formation of a stationary *thermal* grating. The heat release leads to a spatial modulation of the density across the probe volume and thus a modulation in the refractive index of the medium. The rapid changes in the density of the medium due to the heating generate two counter-propagating acoustic waves. The acoustic waves propagate orthogonally with respect to the interference fringes and decay due to acoustic damping and acoustic transit time. The sum of these acoustic waves is a standing wave. The oscillation frequency of the acoustic wave is described as $\hbox {f}_{{osc}}$ = 1/$\hbox {t}_{{osc}}$, where $\hbox {t}_{{osc}}$ is the oscillation period.

For high enough pump laser intensities, a grating may also be formed through electrostriction by non-resonant pump beams. Such a grating is thus called *electrostrictive*. The high electric field of the pump laser beams forces the molecules to move towards the high intensity fringes, which results in density modulation and generation of acoustic waves. The acoustic oscillation frequency of an electrostrictive grating is twice the frequency of a thermal grating^26^.

The oscillation period is related to the fringe spacing, $\Lambda$, and the sound velocity, $\hbox {v}_{{s}}$, as $\hbox {t}_{{osc}}$ = $\Lambda$/$\hbox {v}_{{s}}$. For an ideal gas the sound velocity is given by $\hbox {v}_{{s}}$=$\sqrt{\frac{\kappa }{M}RT}$, where $\kappa$ = $\hbox {C}_{{p}}$/$\hbox {C}_{{v}}$ is the ratio between the heat capacities at constant pressure ($\hbox {C}_{{p}}$) and constant volume ($\hbox {C}_{{v}}$), M is the molar mass, R is the ideal gas constant, and T is the gas temperature. Hence, the following expression is obtained for temperature.

2 $$\begin{aligned} T=\frac{M}{\zeta R}(\Lambda f_{osc} )^{2}. \end{aligned}$$

The dynamics of the laser-induced grating is sensed by a probe laser beam, having a wavelength $\lambda _{probe}$. This beam is usually cross-polarized to the pump laser beams and intersects the grating at the first order Bragg angle, sin($\theta _B$) = $\lambda _{probe}$/2$\Lambda$. Thus, by analyzing the temporal shape, in particular the oscillation frequency $\hbox {f}_{{osc}}$ of the generated signal, it is possible to determine the temperature, given that the major gas composition is known and behaves like an ideal gas.

### Experimental arrangement

**Figure 1:**
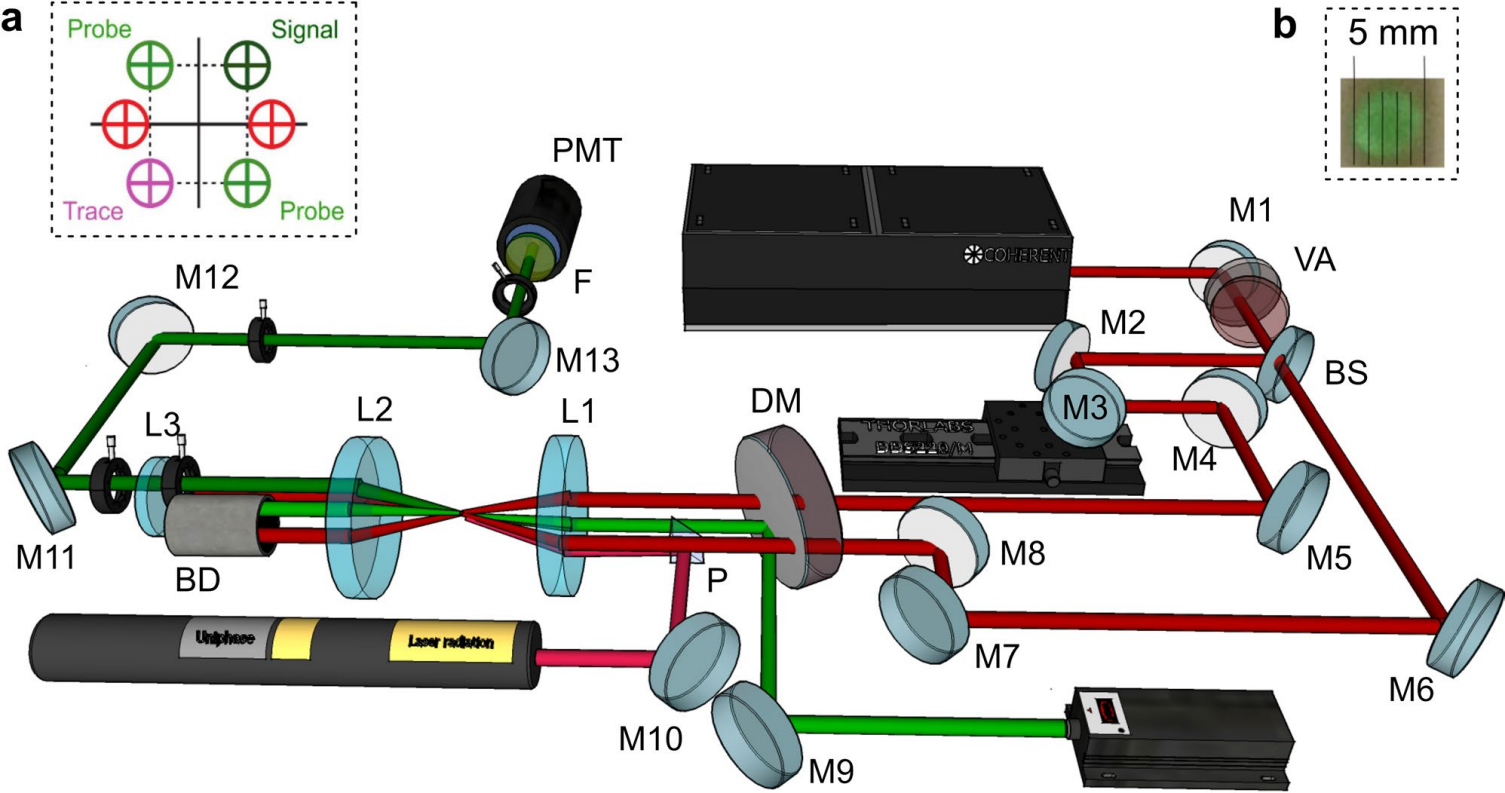
Experimental setup for fs-LIGS; *M1-13* mirrors, *VA* variable attenuator, *BS* beam splitter, *DM* dichroic mirror, *P* prism, *L1-3* spherical lenses, *BD* beam dump, *F* bandpass filter, *PMT* photomultiplier tube. The elements between L2 and L3, between L3 and M11, between M12 and M13, and between M13 and F are iris diaphragms. (**a**) A sketch of a folded BOXCARS configuration used for alignment purposes, (**b**) signal beam observed on a white paper.

Figure 1 shows a schematic outline of the experimental setup. A Ti:Sapphire Chirped Pulsed Amplification system (Hidra Coherent) was used to deliver 125-fs pump laser pulses, 800 nm at 10 Hz repetition rate with a maximum laser energy of 30 mJ per pulse. A variable attenuator for high power linearly polarized lasers, consisting of a thin film Brewster type polarizer and a quartz half-wave plate, was used to control the laser pulse energy, which was kept around 500–700 $\upmu$J in the probe volume for most of the experiments. The laser beam was split into two beams of equal intensities using a beam splitter. An optical delay line consisting of the two mirrors M2 and M3, was used to optimize the temporal overlap of the two pump beams in the probe volume. A linearly polarized continuous wave (cw) laser (MGL-III) served as the probe beam and delivered 350 mW at 532 nm wavelength. This beam, after being reflected by a dichroic mirror was directed to the grating formed by the two 800-nm pump pulses at 1^st^ Bragg angle configuration. A HeNe laser, directed to the probe volume by a prism, was used as a trace laser for alignment of the signal beam. Figure 1a shows the beam configuration used for the alignment of the LIGS setup, where the top probe and signal beams indicate the position of the transmitted and Bragg diffracted beams after intersecting the grating. In this folded BOXCARS configuration the beams were focused and directed into the probe volume by lens L1 (f = 500 mm). The beam waist diameter at the focusing spot was estimated to be 127 $\upmu$m and the length of the measurement volume was estimated to be 5.3 mm. The signal was collected with lenses L2 and L3 and detected using a photomultiplier tube (Hamamatsu, H6780-04), connected to an oscilloscope (LeCroy Waverunner 6100, 1 GHz). The 532 nm LIGS signal could be clearly observed on a white paper with a naked eye (see Fig. 1b). A band-pass filter, centred at 532 nm with FWHM = 4 nm was placed in front of the detector to suppress stray background light. It should be noted that for the strongest signals neutral density filters were used to reduce the signal intensity in order to avoid saturating the detector.

The two fs-laser beams forming a laser induced grating gave rise to fluorescence from $\hbox {N}_{{2}}$ and $\hbox {N}_{2}^{+}$. In order to study this emission spectrally resolved, an f = 500 mm grating spectrograph (Princeton Instruments, Acton SpectraPro 2500) equipped with an intensified CCD camera (ICCD, Princeton Instruments, PIMAX-3) was employed at a position perpendicular to the probe volume. Two gratings, 150 g/mm and 1200 g/mm, were used to resolve the signal with different spectral dispersions. The fluorescence signal was collected using an f = 60 mm, f$\#$ = 1.2 UV condenser lens (B.Halle, Suprasil).

The LIGS temperature measurements were performed in a heated $\hbox {N}_{{2}}$ gas flow. For this purpose, an open T-shaped quartz tube, surrounded by an electric heating wire with isolation, was used. The $\hbox {N}_{{2}}$ gas flow was supplied through the bottom of the tube and the measurement region was located in the middle of its top part, which had an opening for the laser beams to pass through. A thermocouple of type K was inserted into the small opening at the top of the tube and the temperature was measured approximately 2–3 mm above the probe volume. For more details about the heating gas tube, the sketch and the thermocouple position see Sahlberg et al.^27^. By adjusting the current passing through the wire it was possible to heat a 5 l/min $\hbox {N}_{{2}}$ gas flow up to 753 K.

### Electronic structure of $\hbox {N}_{{2}}$ and $\hbox {N}_{2}^{+}$

**Figure 2:**
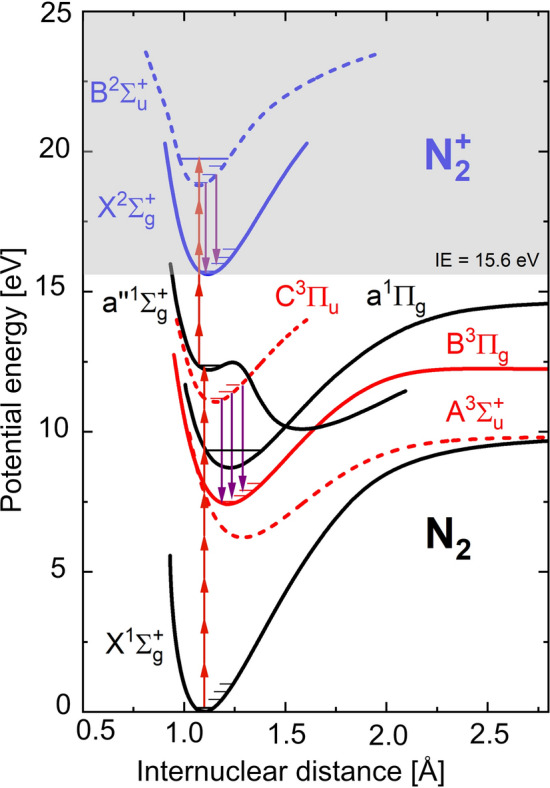
Electronic structure of $\hbox {N}_{{2}}$ and $\hbox {N}_{2}^{+}$, where the ionization energy (IE) is indicated. The energy of an 800 nm photon (1.55 eV) is indicated by a red arrow and the purple arrows show the fluorescence. The potential curves are based on data reported by Western et al.^31^, Ermler et al.^32^, and Singh and Rai^33^.

Potential energy curves of $\hbox {N}_{{2}}$ and $\hbox {N}_{2}^{+}$ relevant for the present study are shown in Fig. 2. For $\hbox {N}_{{2}}$, singlet states are drawn in black, while triplet states are red. The $\hbox {N}_{2}^{+}$ (doublet) states are drawn with blue color. States with even parity, i.e. gerade (g) states, are represented by solid lines, while states with odd parity, i.e. ungerade (u) states, are represented by dashed lines. Since the ground state of $\hbox {N}_{{2}}$ is a singlet, the selection rule $\Delta$S = 0 implies that only singlet states can be directly excited through electric dipole transitions. With 800-nm (1.55 eV) photons it is possible to pump the state $\hbox {a}^{\prime \prime 1}\Sigma _{g}^{+}$ state through 8-photon absorption. As can be seen in Fig. 2 this electronic state is a double well. The double well potential is the result of two $^{^1}\Sigma _{g}^{+}$ states with avoided crossing, one corresponding to the shallow well in the vicinity of an internuclear separation of 1.1 Å and another one, named $^{^1}\Sigma _{g}^{+}$(II), corresponding to the much wider well at larger internuclear separations^28^. If the 800-nm laser intensity is high enough it is possible to ionize the molecule, i.e. excite the $\hbox {X}^{2}\Sigma _{g}^{+}$ ground state of $\hbox {N}_{2}^{+}$, via 3-photon absorption, and even reach the excited $\hbox {B}^{2}\Sigma _{u}^{+}$ state through 5-photon absorption from the $\hbox {a}^{\prime \prime 1}\Sigma _{g}^{+}$ state. Excitation of the $\hbox {B}^{2}\Sigma _{u}^{+}$ state enables fluorescence in the 1$^{st}$ negative system, $\hbox {B}^{2}\Sigma _{u}^{+}$ $\rightarrow$ $\hbox {X}^{2}\Sigma _{g}^{+}$, indicated with purple arrows in Fig. 2. It is also possible to produce $\hbox {N}_{2}^{+}$ through tunnel ionization. For a typical laser intensity of 30 TW/$\hbox {cm}^{2}$ the Keldysh parameter is $\gamma$ = 2.1, i.e. larger than unity, indicating that tunnel ionization is not dominant^29,30^. Since $\gamma$ is rather close to unity, both multi-photon absorption and tunneling contribute to the ionization of $\hbox {N}_{{2}}$, but the former process appears to dominate slightly.

In addition to resonant multi-photon excitation and ionization, the fs-laser pulse can also dissociate the $\hbox {N}_{{2}}$ molecule, resulting in production of N($^{4}$S) atoms. These atoms can then form unstable $\hbox {N}_{{2}}$ ($^{5}\Sigma _{g}^{+}$) through three-body recombination, which subsequently forms $\hbox {N}_{{2}}$ in the $\hbox {B}^{3}\Pi _{g}$ state, which allows fluorescence in the 1^st^ positive system, $\hbox {B}^{3}\Pi _{g}$ $\rightarrow$ $\hbox {A}^{3}\Sigma _{u}^{+}$^34^. In the present study fluorescence in the 2$^{nd}$ positive system, $\hbox {C}^{3}\Pi _{u}$ $\rightarrow$ $\hbox {B}^{3}\Pi _{g}$, was observed, indicated with purple arrows in Fig. 2, meaning that the $\hbox {C}^{3}\Pi _{u}$ state was populated. The mechanism responsible for populating this state is less clear. Direct excitation from the ground state, $\hbox {X}^{1}\Sigma _{g}^{+}$, is spin-forbidden and therefore very unlikely. It has been proposed that the $\hbox {C}^{3}\Pi _{u}$ state can be populated indirectly from the $\hbox {N}_{2}^{+}$ ions, via $\hbox {N}_{2}^{+}$ + $\hbox {N}_{{2}}$ $\rightarrow$ $\hbox {N}_{4}^{+}$ followed by electron capture, $\hbox {N}_{4}^{+}$ + $\hbox {e}^{-}$ $\rightarrow$ $\hbox {N}_{{2}}$($\hbox {C}^{3}\Pi _{u}$) + $\hbox {N}_{{2}}$^35^. Another proposed pathway is multi-photon absorption to any highly excited single state, e.g. the $\hbox {a}^{\prime \prime 1}\Sigma _{g}^{+}$ state, whereupon transfer to the $\hbox {C}^{3}\Pi _{u}$ state can take place through collisional intersystem crossing (ISC)^36^. The mechanism responsible for populating the $\hbox {C}^{3}\Pi _{u}$ state is still debated. Arnold et al.^37^ found in their study that ISC from an intermediate excited singlet state is the dominant formation path, whereas Zheng et al.^38^ in a recent paper, conclude that $\hbox {C}^{3}\Pi _{u}$ is populated through impact excitation of $\hbox {N}_{{2}}$ ($\hbox {X}^{1}\Sigma _{g}^{+}$) by energetic electrons.

### LIGS signal model

The LIGS signals are modelled using the approach described by Kozlov et al.^39,40^. Equation (3) shows the empirical model which describes the time evolution of the LIGS signal. It is obtained by solving the linearized hydrodynamic equations for a spatially modulated variation of the gas density and temperature in the probe volume in the presence of a laser field. The solution is described in the form of a superposition of damped acoustic waves and stationary density modulations during the variation of the refractive index across the fringes of the laser-induced grating.

3 $$\begin{aligned} \begin{aligned} y(t) =&\bigg (M_{i}\Big [\cos ({2\pi f_{osc}t}) \exp {\Big (-\Big (\frac{t}{\tau _{tr}}\Big )^2-\frac{t}{\tau _{a}}\Big )}-\exp {\Big (-\frac{t}{\tau _{th}}\Big )}\Big ] \\&+ M_{f}\Big [\Big (\frac{k_{f}}{1+k_{f}^2}\Big )\sin ({2\pi f_{osc}t})+\Big (\frac{1}{1+k_{f}^2}\Big )\cos ({2\pi f_{osc}t})\exp \Big ({-\Big (\frac{t}{\tau _{tr}}\Big )^2-\frac{t}{\tau _{a}}}\Big ) \\&-\frac{\Big [\exp \Big ({-\frac{t}{\tau _{th}}}\Big )-\exp \Big ({-\frac{t}{\tau _{f}}}\Big )\Big ]}{\tau _{f}\Big (\frac{1}{\tau _{f}}-\frac{1}{\tau _{th}}\Big )}-\Big (\frac{1}{1+k_{f}^2}\Big )\exp {\Big (-\Big (\frac{t}{\tau _{f}}\Big )}\Big ] \\&+ M_{e}\Big [\sin ({2\pi f_{osc}t})\exp \Big ({-\Big (-\frac{t}{\tau _{tr}}\Big )^2 -\frac{t}{\tau _{a}}}\Big )\Big ]\bigg )^2. \end{aligned} \end{aligned}$$

The three factors $\hbox {M}_{{i}}$, $\hbox {M}_{{f}}$, and $\hbox {M}_{{e}}$, are dimensionless coefficients which scale the contributions to the LIGS signal as a result of instantaneous, finite time (fast) energy re-distributions, and electrostriction due to the presence of the strong laser field, respectively. The oscillation frequency of the acoustic wave is defined by $\hbox {f}_{{osc}}$. The acoustic transit time decay constant $\tau _{tr}$ defines the time for the acoustic waves to move out from the probe volume. The acoustic damping time decay constant $\tau _{a}$ characterizes the exponential decay of the acoustic wave due to viscosity, density, and thermal conductivity. With focused laser beams the probe volume is small, which implies that the transit time decay is much faster than the acoustic damping, i.e. $\tau _{tr}$ is smaller than $\tau _{a}$ as can be seen in Table 1, and therefore $1/\tau _{a}$ was neglected in the model. The stationary part of the laser-induced grating decays exponentially due to thermal diffusion and it is determined by the decay constant $\tau _{th}$. The fast relaxation time due to collisions between molecules is defined by the decay constant $\tau _{f}$, and $\hbox {k}_{f} = 2\pi \hbox {f}_{osc}\tau _{f}$. Table 1 summarizes the theoretically calculated parameters for the $\hbox {N}_{{2}}$ molecule at different temperatures for a grating spacing of 23.56 $\upmu$m, which is calculated from Eq. (1).

**Table 1:** Theoretically calculated parameters for $\hbox {N}_{{2}}$ at different temperatures for a grating spacing of 23.56 $\upmu$m.

	295 K	323 K	373 K	473 K	573 K	673 K	753 K
$\hbox {f}_{{osc}}$ (MHz)	14.89	15.57	16.71	18.79	20.62	22.25	23.45
$\tau _{tr}$ ($\upmu$s)	0.128	0.123	0.114	0.101	0.092	0.086	0.081
$\tau _{th}$ ($\upmu$s)	0.657	0.553	0.429	0.286	0.207	0.159	0.133
$\tau _{f}$ (ns)	0.355	0.387	0.448	0.568	0.688	0.808	0.904
$\tau _{a}$ ($\upmu$s)	0.950	0.798	0.622	0.415	0.303	0.232	0.199

## Results

### Visualization of the fs-laser induced grating

**Figure 3:**
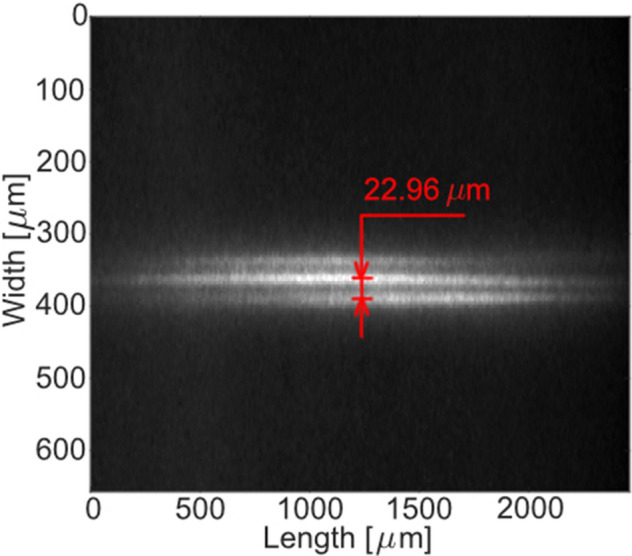
Single-shot fluorescence image of a laser-induced grating in air recorded with a laser pulse energy of 2.8 mJ. The measured grating spacing is 22.96 $\upmu$m.

Figure 3 shows a single-shot fluorescence image of a grating formed in air by the interference of two 125-fs laser pulses. It was recorded with a laser pulse energy of 2.8 mJ and imaged with the ICCD camera. The fluorescence signal from the grating was collected from above the intersection volume with a 20 microscope lens (Olympus Plan N). The field of view of the lens was 2.6 × 2.6 mm (in Fig. 3 the image was cropped along the vertical axis for a better visualization) and the resolution was found to be 50.8 lp/mm according to a 1951 USAF resolution chart. Decreasing the laser pulse energy of the two pump laser beams from 2.8 mJ to 500 $\upmu$J (measured at the probe volume) did not affect the length and the width of the grating. The intensity of the grating drops when approaching lower laser energies of the pump laser beams. At laser pulse energies around 290–300 $\upmu$J the grating is hardly observable. From the recorded image shown in Fig. 3 three grating fringes are observed with a measured fringe spacing of 22.96 $\upmu$m. Changing the polarization of the pump beams before the intersection region did neither affect the intensity nor the shape of the grating. This confirms that the observed luminescence from the grating is due to fluorescence rather than Rayleigh scattering.

### Multi-photon fluorescence spectrum of nitrogen

**Figure 4:**
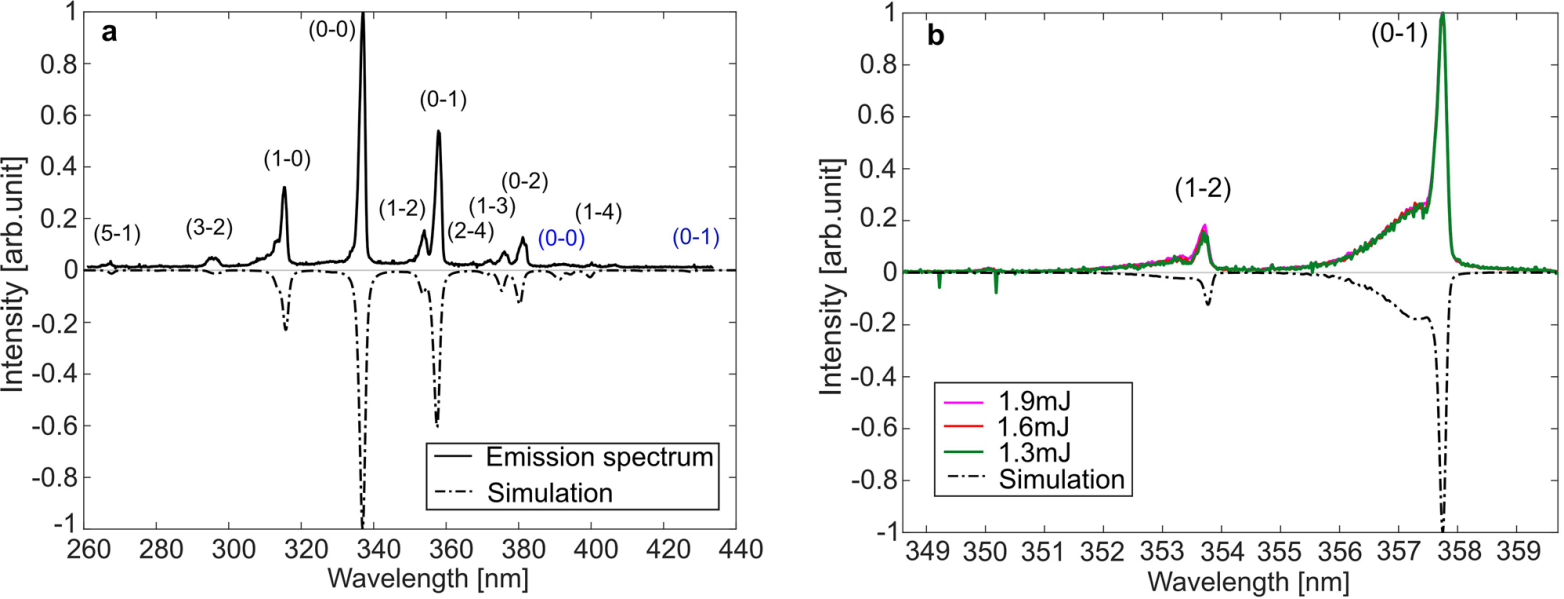
(**a**) Normalized low-resolution $\hbox {N}_{{2}}$ and $\hbox {N}_{2}^{+}$ fluorescence spectra with identified vibrational bands of the transitions $\hbox {C}^{3}\Pi _{u}$ $\rightarrow$ $\hbox {B}^{3}\Pi _{g}$ and $\hbox {B}^{2}\Sigma _{u}^{+}$ $\rightarrow$ $\hbox {X}^{2}\Sigma _{g}^{+}$. The top part (solid black line) shows the emission spectrum recorded in $\hbox {N}_{{2}}$ gas flow at 293 K for a fs-laser pulse energy of 1.9 mJ and the simulated spectrum in PGOPHER is shown in the lower part (dash-dotted black line). (**b**) Normalized high-resolution $\hbox {N}_{{2}}$ fluorescence spectrum showing the vibrational bands of the transition $\hbox {C}^{3}\Pi _{u}$ $\rightarrow$ $\hbox {B}^{3}\Pi _{g}$. The top part shows the emission spectra recorded in $\hbox {N}_{{2}}$ gas flow at 293 K for three different fs-laser pulse energies: 1.3, 1.6 and 1.9 mJ. The lower part (dash-dotted black line) shows the simulated emission spectrum in PGOPHER at 293 K.

Fluorescence spectra of $\hbox {N}_{{2}}$ were measured in a room-temperature flow of nitrogen gas, which was delivered through a 60-mm diameter porous plug. The recorded fluorescence signal is an accumulation of 1000 single-shot signals. In order to produce a detectable $\hbox {N}_{{2}}$ fluorescence signal the laser pulse energy of the two pump beams was kept at 1.9 mJ. The resulting emission spectrum is displayed in the top part as solid black line in Fig. 4a. For this purpose, a low-resolution grating (150 grooves/mm, blaze 300 nm) was used in the spectrograph in order to cover a large spectral region from 260 to 440 nm and show the vibrational bands of $\hbox {N}_{{2}}$ and $\hbox {N}_{2}^{+}$ with unresolved rotational lines. The fluorescence of the 2^nd^ positive system, $\hbox {C}^{3}\Pi _{u}$ $\rightarrow$ $\hbox {B}^{3}\Pi _{g}$, from neutral $\hbox {N}_{{2}}$, and the fluorescence from the vibration bands (0–0 and 0–1) of the 1^st^ negative system, $\hbox {B}^{2}\Sigma _{u}^{+}$ $\rightarrow$ $\hbox {X}^{2}\Sigma _{g}^{+}$, from ionized nitrogen $\hbox {N}_{2}^{+}$ can be observed in the figure.

The ICCD camera used in the experiment has a low sensitivity for detecting the fluorescence in the 1^st^ positive system, $\hbox {B}^{3}\Pi _{g}$ $\rightarrow$ $\hbox {A}^{3}\Sigma _{u}^{+}$, and this band was therefore not investigated in the present work. The dash-dotted black line spectrum, displayed in the lower part, in an intensity-flipped version for clarity, is a theoretical spectrum simulated in PGOPHER^25^ with a rotational temperature $\hbox {T}_{{rot}}$ = 293 K and a vibrational temperature $\hbox {T}_{{vib}}$ = 2500 K. Different vibrational transitions from the $\hbox {C}^{3}\Pi _{u}$ to $\hbox {B}^{3}\Pi _{g}$ state are marked with black brackets, showing that the strongest transition is (0–0) at 337 nm. The two weak bands, (0–0) at 391 nm and (0–1) at 428 nm, in the first negative system, $\hbox {B}^{2}\Sigma _{u}^{+}$ $\rightarrow$ $\hbox {X}^{2}\Sigma _{g}^{+}$, of $\hbox {N}_{2}^{+}$ are marked with blue brackets. The Gaussian and Lorentzian contributions to the linewidths of the vibrational bands in the simulated spectrum have been adjusted to match the experimental data. All emission bands are well predicted by the simulated spectrum. The observed emission spectrum confirms that the $\hbox {C}^{3}\Pi _{u}$ state in $\hbox {N}_{{2}}$ and the $\hbox {B}^{2}\Sigma _{u}^{+}$ state in $\hbox {N}_{2}^{+}$ have been populated by the 800-nm laser pulses, which requires multi-photon excitation (see Fig. 2).

Figure 4b shows the normalized emission spectra of the 2^nd^ positive system, $\hbox {C}^{3}\Pi _{u}$ $\rightarrow$ $\hbox {B}^{3}\Pi _{g}$, in the spectral region 350–360 nm recorded at three different pulse energies (1.3, 1.6 and 1.9 mJ) using the high-resolution grating (1200 grooves/mm). The dash-dotted black line spectrum, in the lower part of the figure, which is simulated with PGOPHER for a rotational temperature of 293 K, i.e. room temperature, agrees very well with the experimental spectra. As can be seen, the rotational “tails” of the ($\hbox {v}^{\prime }=0\rightarrow \hbox {v}^{\prime \prime }$=1) and ($\hbox {v}^{\prime }=1\rightarrow \hbox {v}^{\prime \prime }$=2) transitions are observable and it is clear that the shape of these remains the same for all three laser-pulse energies, meaning that there is no significant energy (heat) deposited to the gas. This result indicates that laser heating is insignificant for laser pulse energies up to 1.9 mJ.

**Figure 5:**
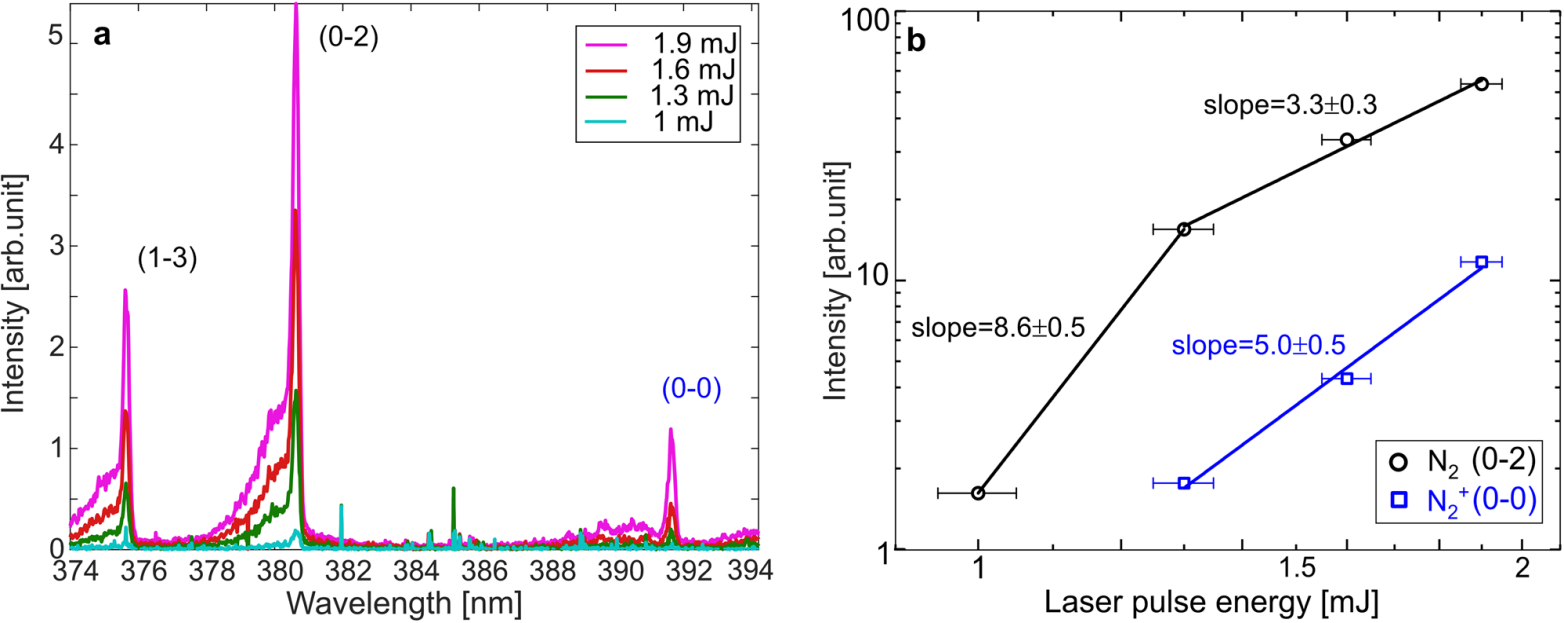
(**a**) High-resolution $\hbox {N}_{{2}}$ and $\hbox {N}_{2}^{+}$ fluorescence spectra with vibrational bands of the transitions $\hbox {C}^{3}\Pi _{u}$ $\rightarrow$ $\hbox {B}^{3}\Pi _{g}$ and $\hbox {B}^{2}\Sigma _{u}^{+}$ $\rightarrow$ $\hbox {X}^{2}\Sigma _{g}^{+}$ recorded at different pulse energies. (**b**) Power dependence of the $\hbox {N}_{{2}}$ fluorescence signal for $\hbox {C}^{3}\Pi _{u}$ $\rightarrow$ $\hbox {B}^{3}\Pi _{g}$ (0–2) transition and $\hbox {N}_{2}^{+}$ fluorescence signal for $\hbox {B}^{2}\Sigma _{u}^{+}$ $\rightarrow$ $\hbox {X}^{2}\Sigma _{g}^{+}$ (0–0) transition.

Figure 5a shows emission spectra of the 2^nd^ positive system, $\hbox {C}^{3}\Pi _{u}$ $\rightarrow$ $\hbox {B}^{3}\Pi _{g}$ and the 1^st^ negative system, $\hbox {B}^{2}\Sigma _{u}^{+}$ $\rightarrow$ $\hbox {X}^{2}\Sigma _{g}^{+}$ in the spectral region 374–394 nm recorded at different laser pulse energies ranging between 1–1.9 mJ. The weak peak around 391 nm corresponds to the (0–0) vibrational band in the 1^st^ negative system, $\hbox {B}^{2}\Sigma _{u}^{+}$ $\rightarrow$ $\hbox {X}^{2}\Sigma _{g}^{+}$, of $\hbox {N}_{2}^{+}$. This peak is observable for pulse energies 1.3 mJ and above, which indicates that ionization should be insignificant for pulse energies below 1.3 mJ. This is an important observation since the laser energies used to record LIGS signals in the current study are around 500–700 $\upmu$J.

Formation of a thermal grating requires energy to be stored in excited states of the target molecule. To investigate the possible paths for such excitation, emission spectra covering two vibrational bands in the 2^nd^ positive system of $\hbox {N}_{{2}}$ and the (0–0) band in the 1^st^ negative system of $\hbox {N}_{2}^{+}$ were recorded for four different laser pulse energies, as shown in Fig. 5a. Figure 5b shows a logarithmic diagram where the intensities of the (0–2) peak of $\hbox {N}_{{2}}$ and the (0–0) peak of $\hbox {N}_{2}^{+}$ have been plotted against the laser pulse energy. The horizontal error bars indicate the pulse-to-pulse uncertainty of the laser energy, which was estimated to be ± 0.05 mJ. Although consisting of a limited number of data points, the slopes of the signal dependencies suggest that the fluorescence emitted in the $\hbox {N}_{{2}}$ 2^nd^ positive system follows an 8–9 photon absorption process for pulse energies between 1 and 1.3 mJ. The dependence then becomes much weaker, which indicates a 3-photon absorption process for pulse energies ranging from 1.3 to 1.9 mJ, where ionization of $\hbox {N}_{{2}}$ is more feasible to happen. As can be seen from Fig. 2, this signal behavior is rather well supported by the hypothesized 8-photon excitation from $\hbox {X}^{1}\Sigma _{g}^{+}$ to $\hbox {a}^{\prime \prime 1}\Sigma _{g}^{+}$ state (see the previous section on the electronic structure of $\hbox {N}_{{2}}$ and $\hbox {N}_{2}^{+}$), from which the $\hbox {B}^{2}\Sigma _{u}^{+}$ state can be reached by a 5-photon process. This suggests an $\hbox {E}_{pulse}^{8}$ dependence for low pulse energies, which turns gradually to an $\hbox {E}_{pulse}^{8-5}$ = $\hbox {E}_{pulse}^{3}$ dependence with increasing pulse energy. The slope of the signal dependence for the $\hbox {N}_{2}^{+}$ fluorescence in the first negative system is approximately 5 ± 0.5, suggesting a 5-photon process.

### Analysis of LIGS signals

**Figure 6:**
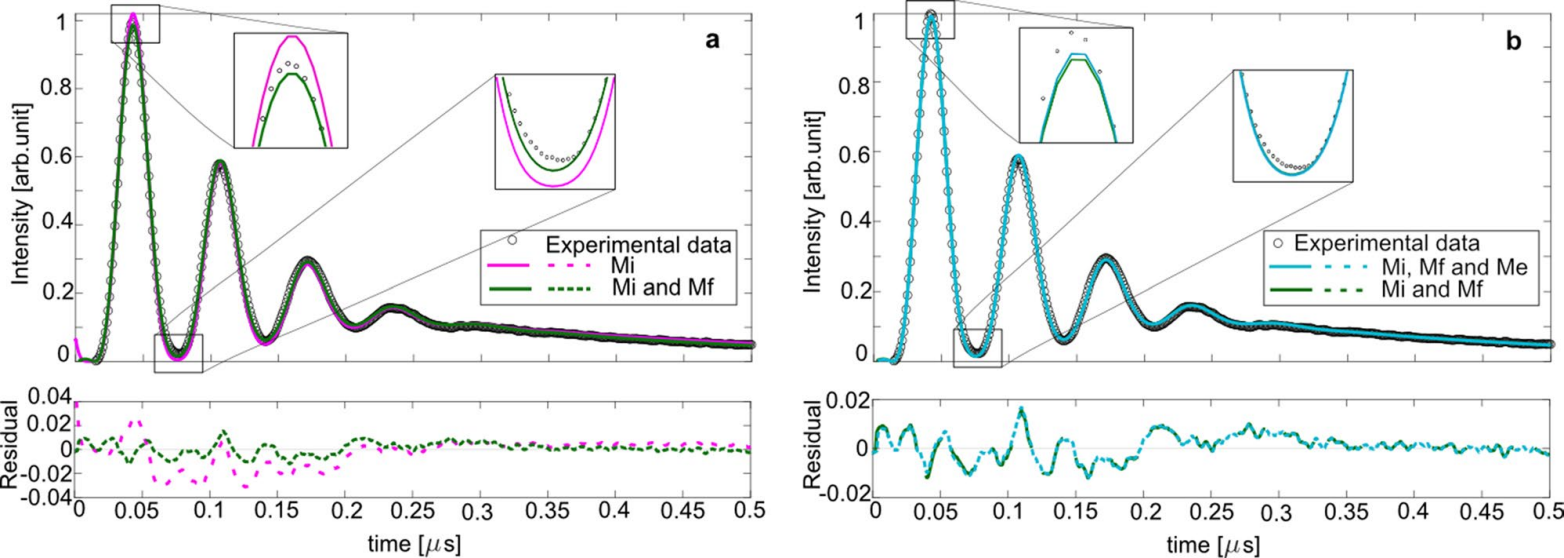
Measured, at 700 $\upmu$J laser pulse energy, and modelled LIGS signals of $\hbox {N}_{{2}}$ at 323 K . The modelled signals are based on Eq. (3). (**a**) Pink curve corresponds to the model containing only the instantaneous term ($\hbox {M}_{{i}}$) and the green curve is the model where both the instantaneous and finite terms ($\hbox {M}_{{i}}$ and $\hbox {M}_{{f}}$) are included. (**b**) Green curve corresponds to the model containing both the instantaneous and finite terms ($\hbox {M}_{{i}}$ and $\hbox {M}_{{f}}$), while the blue curve shows the model containing the instantaneous, finite and electrostrictive terms ($\hbox {M}_{{i}}$, $\hbox {M}_{{f}}$ and $\hbox {M}_{{e}}$).

A theoretical model based on Eq. (3) and least-squares fitting routine was developed to predict the experimental LIGS signal recorded in $\hbox {N}_{{2}}$ gas flow at 323 K. Moreover, the scaling factors ($\hbox {M}_{{i}}$, $\hbox {M}_{{f}}$ and $\hbox {M}_{{e}}$) were varied in order to investigate the weight of each of them on the model. The main challenge of this approach is to find a good initial guess of the parameters (see the constants in Table 1). Figure 6 shows measured (circles) and modeled LIGS signals of $\hbox {N}_{{2}}$ at 323 K at 700 $\upmu$J laser pulse energy. The model predicts the peak positions very well, meaning that the acoustic frequency $\hbox {f}_{{osc}}$ is well chosen. However, a closer look at the fitted curves reveals some discrepancies. Let us first assume that the signal is only generated by the “instantaneous” spatially modulated heat exchange, i.e. $\hbox {M}_{{f}}$ and $\hbox {M}_{{e}}$ in Eq. (3) are set to zero. This thermal process takes place when $\tau$ $\gg$ $\hbox {T}_{{osc}}$/2$\pi$, where $\tau$ represents time constants $\tau _{tr}$ and $\tau _{th}$ presented in the Eq. (3)^39^. The pink curve in Fig. 6a represents the model with only the instantaneous term ($\hbox {M}_{{i}}$) included. This model shows a poor agreement with experimentally measured curve. The first peak does not fit well since it is above the experimental data and the first valley of the fit is below the experimentally measured curve (see the magnified inserts). It seems that a sole contribution from the instantaneous heat transfer is insufficient for accurate prediction of the signal. Therefore, the finite heat deposition term (the term scaling with $\hbox {M}_{{f}}$) needs to be included in the model. The green curve in Fig. 6a represents the model where both the instantaneous ($\hbox {M}_{{i}}$) and finite term ($\hbox {M}_{{f}}$) are included. The residuals, plotted in the lower panel of Fig. 6a, reveal that there is a small difference between the two modeled signals. It should be noted that the initial guess for the finite decay constant was set to 0.38 ns (see Table 1 for 323 K) based on a quenching rate constant of k = 1.15 $\times$ 10$^{-11}$ $\hbox {cm}^{3}$ $\hbox {s}^{-1}$ for the 2^nd^ positive band $\hbox {C}^{3}\Pi _{u}$ $\rightarrow$ $\hbox {B}^{3}\Pi _{g}$^41^. However, there are few possible collisional relaxation mechanisms that can contribute to the finite decay of the observed signal. To assume that quenching, and solely in the $\hbox {C}^{3}\Pi _{u}$ $\rightarrow$ $\hbox {B}^{3}\Pi _{g}$ band system, is the only contribution to $\tau _{f}$ is of course a major simplification. However, an investigation of possible molecular energy transfer processes that generate heat for the formation of the thermal grating is not within the scope of the present work. Nevertheless, in the model it was assumed, as a first approximation, that the energy conversion is well described by a single rate, i.e. a finite relaxation time $\tau _{f}$ = 0.38 ns for 323 K. This assumption is justified by the fact that the modeled signal shape (green curve in Fig. 6a) predicts the measured signal very well.

Two modeled signals are shown in Fig. 6b, where the first one contains both the instantaneous ($\hbox {M}_{{i}}$) and finite ($\hbox {M}_{{f}}$) contribution (green curve), while the second one still includes both of these terms but also an electrostrictive ($\hbox {M}_{{e}}$) contribution (blue curve). It is difficult to observe any difference between two curves, even from the corresponding residuals shown in the lower panel of Fig. 6b, which means that the electrostriction can be negligible. Including instantaneous and finite energy redistribution is, in our experiments, already sufficient for a good prediction of the signal if the laser energies are kept below 500–700 $\upmu$J. However, it should be noted, that for higher laser energies grating becomes distorted and an electrostrictive ($\hbox {M}_{{e}}$) term can have an influence on the modeling, even if a pure thermal signal is analyzed.

**Figure 7:**
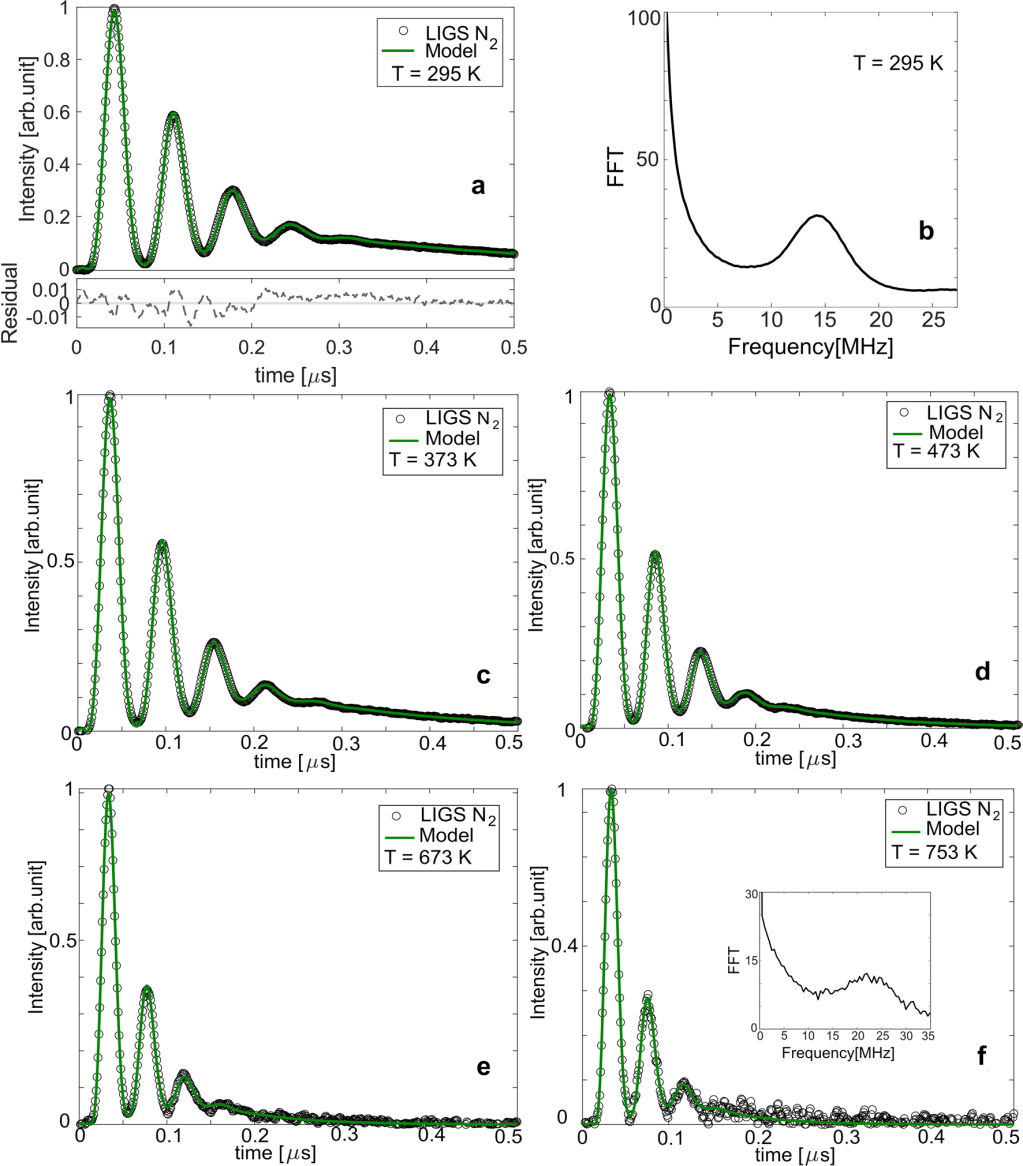
LITGS signals recorded in $\hbox {N}_{{2}}$ gas flow at five different temperatures at 700 $\upmu$J laser pulse energy. The different panels correspond to signals recorded at (**a**) 295 K, (**c**) 373 K, (**d**) 473 K, (**e**) 673 K, and (**f**) 753 K. (**b**) Fourier transform of the LITGS signal in (**a**) for 295 K.

Figure 7 shows thermal LIGS (LITGS) signals (circles) recorded in $\hbox {N}_{{2}}$ gas flow at five different temperatures, ranging from 295 to 753 K at 700 $\upmu$J laser pulse energy. Each signal is an average value of 100 single-shot LITGS signals. The recorded LITGS signals for temperatures up to 473 K had strong intensity, and thus, neutral density filters were used in front of the PMT to avoid saturation of the detector. However, this could be avoided by reducing the laser pulse energy down to 100 $\upmu$J (4.5 TW/$\hbox {cm}^{2}$) while a sufficient signal-to-noise ratio (SNR) was maintained.

The model was fitted to the measured data (circles) with the aim to extract the oscillation frequency ($\hbox {f}_{{osc}}$). The fitted curves are represented in green colour. The dashed black curve in the lower panel of Fig. 7a shows the difference between the experimental data and the fitted curve. The residuals between the experimental LITGS signal and the fitted model were investigated for all temperature cases in order to ensure a reliable fit, but is here only shown for 295 K. One can notice that with increasing temperature the number of oscillation peaks decreases, and the thermal decay increases ($\tau _{th}$ decreases). This is in a good agreement with the theoretical calculations as can be seen from Table 1.

From the fitted model, the oscillation frequency was extracted from each experimental signal. The temperatures were then determined using Eq. (2). Another approach to retrieve the oscillation frequency is to analyze the Fourier transform of the LITGS signal, as shown in Fig. 7b. However, at high temperatures the frequency peak in the Fourier transform (inset in Fig. 7f) becomes broader due low number of resolved oscillation peaks and hence, the uncertainty in determining the oscillation frequency increases.

**Figure 8:**
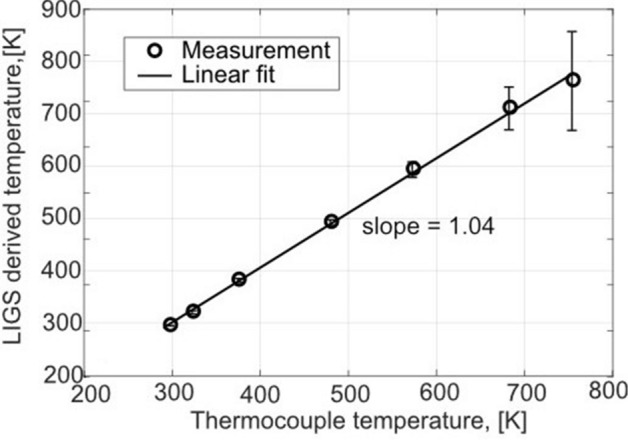
Correlation between the LITGS derived temperatures and the temperatures measured by thermocouple. The measurements were performed in a $\hbox {N}_{{2}}$ gas flow at 700 $\upmu$J laser pulse energy.

Figure 8 shows a comparison between the evaluated temperatures and the temperatures measured with a thermocouple. The vertical error bars indicate the standard deviations for 100 single-shot LITGS signals for each temperature. In total seven temperatures have been measured and the uncertainty in the derived LIGS temperature for all investigated gas temperatures was: $296.1\pm 1\ \hbox {K}$, $321.7\pm 3\ \mathrm{K}$, $382.8\pm 5.8\ \mathrm{K}$, $493\pm 5.2\ \mathrm{K}$, $594\pm 14.7\ \mathrm{K}$, $710.9\pm 40.8\ \mathrm{K}$, $763.2\pm 94.2\ \mathrm{K}$.

The thermocouple was located 2–3 mm away from the intersection point of the two laser beams. This was the closest position possible without intruding the measurement. The thermocouple error shows the variation of the temperature during the experimental data collection for each temperature. This error was observed to be 1$\%$ of the investigated gas temperatures, which has a negligible impact on the conclusions. It is clear that the ideal linear fit between the calculated LITGS temperatures and the measured ones is true within the measurement uncertainty range. The increasing uncertainty with increasing temperature arises from the uncertainty in precisely determining the oscillation frequency due to the decreasing number of resolved oscillation peaks of the LITGS signals (compare Fig. 7f which has 3 peaks and Fig. 7a which has 4 peaks). With increasing temperature, the decay of the LITGS signal due to thermal diffusion is faster. This means that the grating dissolves faster compared to at room temperature. The uncertainty is also affected by the signal intensity, since it affects how many oscillation peaks that can be identified. Nevertheless, the linear relation between the LIGS derived temperature and the thermocouple temperature shows the functionality of LIGS as a thermometry technique within the investigated temperature range.

**Figure 9:**
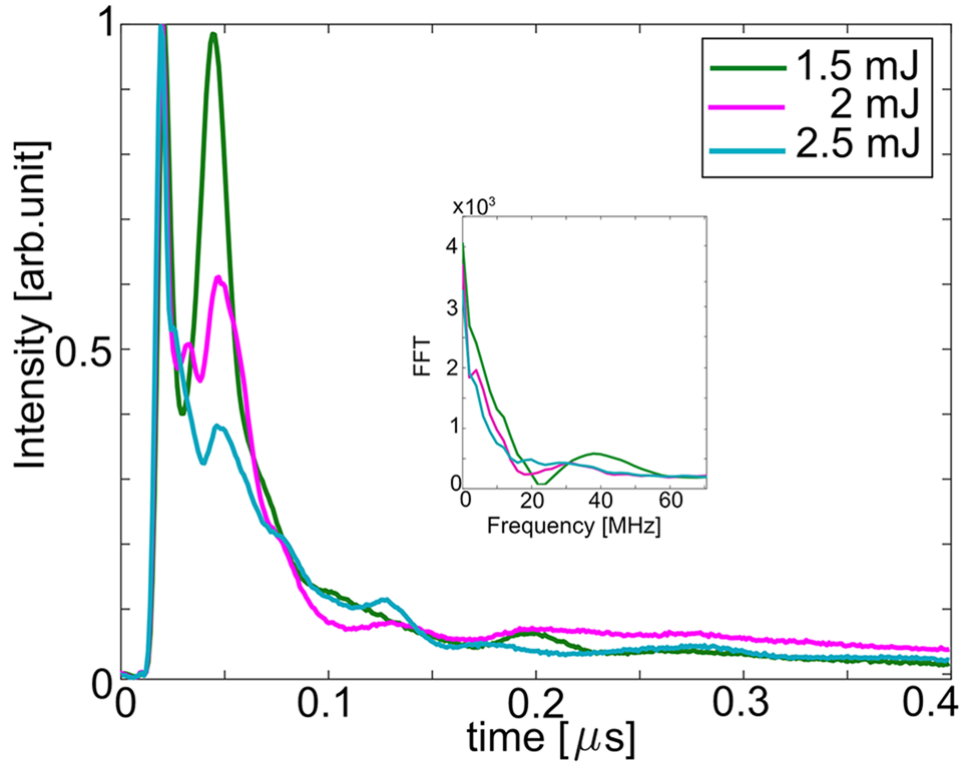
Recorded single-shot LIGS signals in $\hbox {N}_{{2}}$ at fs-laser pulse energies 1.5 mJ (green curve), 2 mJ (pink curve) and 2.9 mJ (blue curve) respectively. The inset shows the Fourier analysis of the signals at these energies.

For laser pulse energies higher than 700 $\upmu$J, the shape of the LIGS signal changes profoundly, in which the oscillations are less regular and shifted. The reason for this transition is that the gas gets heated and weakly ionized resulting in a plasma grating^42^ (see Fig. 9). The thermal grating becomes extremely unstable due to the plasma formation. From 1 mJ pulse energy (63 TW/$\hbox {cm}^{2}$ ) the $\hbox {N}_{2}^{+}$ (0–0) band shown in Fig. 5a is observable, meaning that the ionization threshold is reached. The signals presented in Fig. 9 were recorded in $\hbox {N}_{{2}}$ gas flow at room temperature for three different pulse energies: 1.5, 2, and 2.9 mJ, respectively. As can be seen in the figure, the typical periodic modulation of the signal has disappeared, reflecting that the hydrodynamics in a plasma grating is vastly different compared to a thermal grating^42^. The results also show that the signal intensity essentially remains the same for the investigated laser pulse energies, whereas their signal shapes change irregularly.

In addition, by increasing the energy the fs-LIGS signal becomes even more unstable due to the highly nonlinear phenomena involved in the plasma dynamics. Moreover, the plasma lifetime is typically on the order of 100 ps^42^, but the lifetime of the fs-LIGS signal is on the ns-scale, which suggests that the hydrodynamic process following the plasma takes a longer time. It is clear that accurate thermometry based on the present model requires that the laser pulse energy is kept below $\sim$ 700 $\upmu$J. Diagnostic concept at higher pulse energies could potentially be developed, but further investigations, including both experimental and theoretical studies, are required in order to understand the underlying mechanism of a plasma-grating-meditated fs-LIGS signal.

## Discussion

It has been demonstrated in this work that a multi-photon excitation process is required in order to excite $\hbox {N}_{{2}}$ using the 800-nm wavelength provided by a powerful femtosecond Ti:Sapphire laser. However, it might be possible to achieve substantially stronger signals with 2-photon excitation in the UV regime using lower laser energies. For example, 2-photon excitation from $\hbox {X}^{1}\Sigma _{g}^{+}$ to $\hbox {a}^{1}\Pi _{g}$ would require a wavelength around 267 nm, while 2-photon excitation from $\hbox {X}^{1}\Sigma _{g}^{+}$ to $\hbox {a}^{\prime \prime 1}\Sigma _{g}^{+}$ corresponds to a laser wavelength of 200 nm. The former alternative would require a tunable laser, while the latter excitation scheme could be realized by quadrupling a Ti:Sapphire laser. Kaminski and Dreier^43^ have performed a two-photon-induced polarization spectroscopy of molecular nitrogen from $\hbox {X}^{1}\Sigma _{g}^{+}$ to $\hbox {a}^{1}\Pi _{g}$ at around 283 nm wavelength at atmospheric and elevated pressures.

For thermometry with LIGS it is crucial that no significant laser heating takes place. Our results indicate that this is not an issue as long as the laser pulse energy is kept below 700 $\upmu$J. In a previous work on thermometry with FLEET^44^, also based on excitation of nitrogen with 800-nm fs-laser pulses, it is reported that a reasonable estimate is that 1% of the pulse energy is converted into heat in the focal volume. In our case with 700 $\upmu$J laser pulse energy a rough estimation suggests that laser heating would increase the temperature by less than 10 K.

**Figure 10:**
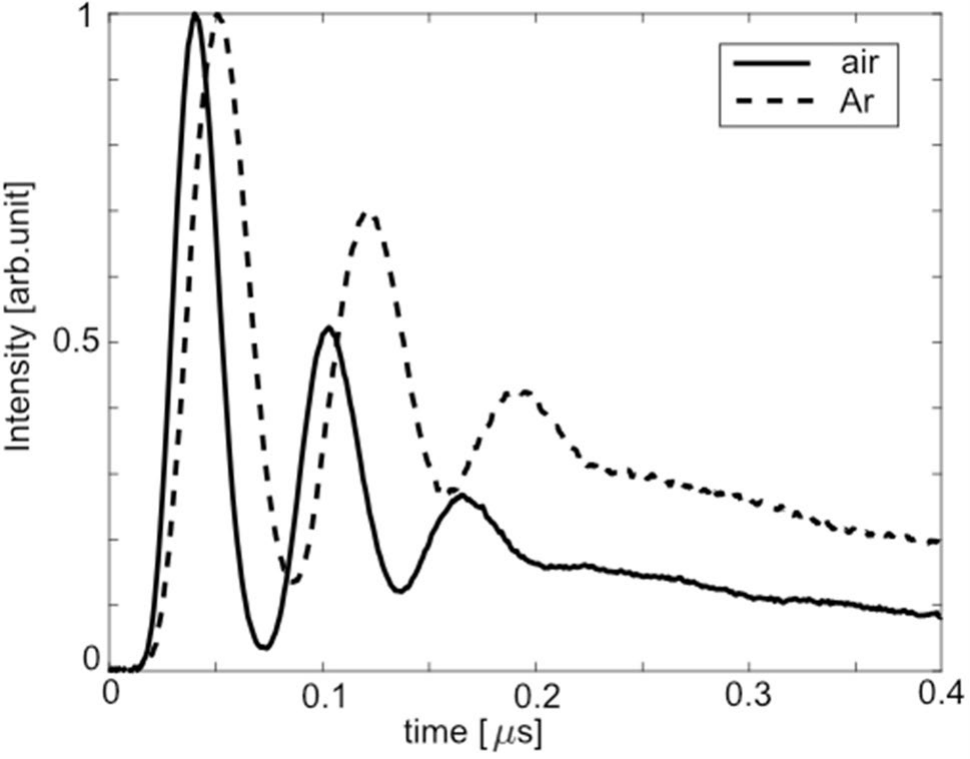
Normalized fs-LIGS signals of air and argon (Ar) recorded in a gas flow of respective gas at the laser energy 500 $\upmu$J (30 TW/$\hbox {cm}^{2}$).

The fs-LIGS technique is not restricted to nitrogen, it can be employed in other gases and mixtures as well. Thus, two more gases, air and argon (Ar), have been studied. The excitation and ionization pathways of Ar are different compared to $\hbox {N}_{{2}}$. The lowest excited state of argon is lying 12.41 eV above the ground state. This energy difference corresponds to 8 photons of 800 nm ($\sim$ 1.55 eV). However, this state is not accessible since the transition is not allowed by the selection rules. There are several other excitation alternatives, which require 9 or 10 photons but they are also ruled out by the selection rules. The only remaining alternative is multi-photon ionization, which requires at least 11 photons. In order to support this hypothesis, a thorough spectroscopic study of the emission spectrum of argon is required, which is outside the scope of the current work. It was found that the LIGS signal from Ar is in general weaker than signal formed in air ($\sim$ 100 times) and $\hbox {N}_{{2}}$ gas flow ($\sim$ 10 times). Figure 10 shows normalized fs-LIGS signals recorded at room temperature in gas flows of argon and air, respectively. As can be seen, the signal recorded in air exhibits a higher oscillation frequency than the one observed for argon, due to the difference in speed of sound for these gases.

Previously, LIGS has shown advantageous application at elevated pressures^9^. This makes it interesting to explore the fs-LIGS technique at elevated pressures, starting in a high-pressure cell with variable temperature. Such a study could potentially pave the way for practical applications in gas turbines and high-pressure burners. It could be of interest to pursue the technique also at sub-atmospheric pressures by using a bit higher fs-laser pulse energies for the excitation.

## Conclusions

In this work, we have demonstrated the formation of a laser-induced thermal grating in $\hbox {N}_{{2}}$ gas flow using an 800 nm fs-laser. The presence of the grating has been visualized by imaging the fluorescence from $\hbox {N}_{{2}}$ with an ICCD camera. The feasibility of using fs-LIGS has been validated for thermometry. Strong single-shot LITGS signals have been observed for temperatures ranging from 295 to 753 K. A simulation model has been fitted to the recorded LITGS signals in order to retrieve the oscillation frequency and determine the temperatures. The measured temperatures have shown a good agreement with the values measured by a thermocouple, making fs-LIGS a potential diagnostic technique for the temperature measurements in gases.

A major finding of this study is that the high peak power and wide spectral linewidth of fs-laser pulses allow generation of LITGS signals from nitrogen through multi-photon excitation. This eliminates the need for a laser tuned to a specific frequency, matching an atomic/molecular resonance frequency, which is the case when ns pulses are used for excitation. We have performed fluorescence studies in order to investigate the possible multi-photon absorption pathways for $\hbox {N}_{{2}}$ at 800 nm wavelength. The 2^nd^ positive system, $\hbox {C}^{3}\Pi _{u}$ $\rightarrow$ $\hbox {B}^{3}\Pi _{g}$, of $\hbox {N}_{{2}}$ and the (0–0) band in the 1$^{st}$ negative system, $\hbox {B}^{2}\Sigma _{u}^{+}$ $\rightarrow$ $\hbox {X}^{2}\Sigma _{g}^{+}$, of $\hbox {N}_{2}^{+}$ have been examined in the spectroscopic fluorescence studies. It has been found that with 800-nm laser pulses, 8 photons are required to populate the $\hbox {C}^{3}\Pi _{u}$ state of $\hbox {N}_{{2}}$. Even though this work has been focused on the multi-photon excitation at 800 nm wavelength for LIGS signals it might be possible to achieve substantially stronger signals for $\hbox {N}_{{2}}$ with 2-photon excitation in the UV regime.

In addition to nitrogen, we also observed LITGS signals from air and argon, respectively, suggesting that the present fs-LIGS concept could be applied also in mixtures containing these gases.

We have found that increasing the laser pulse energies above the ionization threshold (100 TW/$\hbox {cm}^{2}$ for the present study) evokes the formation of a plasma grating, which seems not to be suitable for temperature measurements.

With the promising results from the present study, we firmly belive that fs-LIGS has a strong potential for applications in harsh environments, such as high-pressure boilers and gas turbines, and we intend to explore this in the near future.
